# Influence of Public Engagement with Science on Scientific Information Literacy During the COVID‑19 Pandemic: Empirical Evidence from College Students in China

**DOI:** 10.1007/s11191-021-00261-8

**Published:** 2021-08-30

**Authors:** Chao Gu, Yi Feng

**Affiliations:** 1 Department of History of Science, Technology and Medicine, Peking University, Beijing, China; 2 Mental Health Center, Central University of Finance and Economics, No.39 South College Road, Haidian District, 100081 Beijing, China; 3 Faculty of Psychology, Beijing Normal University, Beijing, China

## Abstract

During the COVID-19 pandemic, we are fighting not only the novel coronavirus, but also the “infodemic” induced by the pandemic. Therefore, it is urgent to explore approaches for enhancing individual immunity against science-related misinformation. We conducted a cross-sectional study to examine the relationship between public engagement with science (PES) and scientific information literacy (SIL) during the COVID-19 pandemic from college students (*N* = 8075) in China. The results showed that there was a significant difference between attitudes toward and activities of PES. More importantly, both PES activities and PES attitudes were found positively associated with SIL, especially the PES attitudes. The empirical study is significant in demonstrating the predictive effect of PES on individual ability to recognize science-related misinformation, which is crucial for mitigating harm from the “infodemic.” Our study indicated that other than a science communication model in order to restore public trust in science, PES is promising to be incorporated into informal science education to facilitate individual SIL.

## Introduction

Human society has been experiencing considerable challenges in public health, politics, and economics due to the rapid spread of the coronavirus disease 2019 (COVID-19) worldwide. From severe acute respiratory syndrome (SARS), and Ebola to COVID-19, epidemics have never been a simple medical or epidemiological concern but rather a comprehensive social governance issue causing major public crises. Because science is deeply embedded in the modern society, the epidemic and associated public crises cannot be eliminated without the help of science.

Thus far, the interaction between science and society is crucial during the pandemic. On the one hand, there are several salient disputes over scientific protective actions and development in the scientific community which influence public perspectives on COVID-19 policy (Hart et al., 2020), such as lockdowns, wearing facemasks, social distancing, and vaccinations. On the other hand, science communication has been a preventive tool in terms of the “infodemic” (Matta, 2020), which has helped reduce the rapid spread of questionable information. The World Health Organization (WHO) defines an “infodemic” as an “overabundance of information—some accurate and some not—occurring during an epidemic” (WHO, 2020). There is considerable misinformation (Galvão, 2020), including a narrative stating that COVID-19 is manmade or was created in a laboratory in Wuhan (Bolsen et al., 2020). In particular, trivializing the risks of COVID-19, questioning the effectiveness of control measures, promoting unproven treatments (e.g., hydroxychloroquine), and contradicting public health experts erodes trust in science and misleads people regarding the effective precautions. Now, governments and the public are fighting not only a pandemic but also a co-evolving “infodemic” (Gallotti et al., 2020). However, limited attention has been paid to enhance individual cognitive immunity against misinformation during the COVID-19 pandemic in the context of informal science education (ISE).This article is an effort to fill this gap. We examined empirically the relationship between public engagement with science (PES) and individual scientific information literacy (SIL) during the COVID-19 pandemic in China. Based on our analysis, although a significant difference was noted between PES attitudes and activities in China, they both promoted individual SIL. The results underscored the importance of PES as a useful complement to the current ISE toolbox in combating the “infodemic.”

## Framework

### PES in China

Overall, a gradual and incomplete shift is noted from “public understanding of science” seen as the “deficit model” toward “public engagement in science and technology” (Holden, 2002) seen as the “dialog model” (Davies & Horst, 2016), which primarily indicates the inclusion of the public into discussions with scientists regarding crucial science-related issues. The aim of public engagement is to facilitate the exchange of information, knowledge, perspectives, and preferences among groups that vary in expertise, power, and values as well as to find their common ground (National Academies of Sciences & Medicine, 2017). Furthermore, PES is framed as a multi-directional dialog among people and mutual learning by publics and scientists that allow all the participants to develop more nuanced understandings of scientific issues. PES thus fits in with goals of ISE (McCallie et al., 2009).

One of the most active and exciting research agendas in recent years for PES is analyzing the contours of “PES with Chinese characteristics” with distinctive models and approaches (Stilgoe et al., 2014). Jia and Liu (2014) concluded that the effort to popularize science based on the deficit model cannot meet the diversifying demands on science in Chinese society. To cope with the challenge, the current unidirectional model of science popularization must be replaced with more engagement practices that feature open and equal public dialog and debate while “the country’s first pilot consensus conference” has already been held. However, governments tend to prevent the public from participating in social management because of concerns about the erosion of authority, which has made PES in China a low priority (Xu et al., 2015). However, several empirical studies have shown that PES is appearing as an element in certain public issues in China. For example, activists use science communication as a way of protesting the current political, medical, and cultural structures around the use and funding of Traditional Chinese Medicine in China (Zhu & Horst, 2019). PES is also crucial for improving the acceptance of some controversial technologies and issues such as nuclear energy development. Previous studies revealed that PES in China was historically at a low level, and demonstrated the positive effect of PES on public acceptance of nuclear energy (Wang et al., 2019). Moreover, increasing numbers of Chinese citizens have participated in the open discussion and public debates about genetically modified organisms (GMO), in accord with the rapid growth of Chinese native social media (Xu et al., 2018).

Considering the complex and ambiguous nature of PES in China because of its specific social context, previous studies have not referred to the discrepancy between the rising enthusiasm of participation in science and low level of actual participation of Chinese citizens. As official Chinese science communicators worry that organizing or being involved in public debates could bring political risks, and it is not necessary for Chinese scientific community to win public support due to China’s top-down approaches in policy making and funding decisions (Jia & Liu, 2014), PES activities in China probably do not keep pace with PES attitudes. Therefore, the first hypothesis on PES is proposed as follows:

#### Hypothesis 1 (H1)

PES activities will be significantly lower than PES attitudes in China.

### SIL

In the battle against the “infodemic,” individuals must achieve societal immunity against misinformation (Linden et al., 2017). Recent attempts to combat widespread misinformation have primarily focused on empowering individuals to recognize misinformation (Scheufele & Krause, 2019). That is, individuals should be equipped with the skillset to separate facts from falsehoods, distinguish dated, biased, or exploitative sources and select intelligently when overwhelmed by an abundance of information (Livingstone, 2004). Several terminologies—such as news and media literacy—describe “the ability to access, analyze, evaluate, and create messages in a variety of forms” while the specific “evaluation” skill is typically conceptualized as information literacy (Potter, 2010). In theory, all these types of literacies should be crucial in combating misinformation with critical thinking. However, some researchers have demonstrated that information literacy—but not other literacies—significantly increased the likelihood of identifying fake news (Jones-Jang et al., 2019). Furthermore, some specific types of media or information literacies in a changing media environment are emerging. For example, eHealth literacy is defined as the ability to seek, find, and understand health information from electronic sources to subsequently make appropriate health decisions (Swire-Thompson & Lazer, 2020). Nevertheless, limited research has been conducted on such literacy regarding the evaluation of scientific information. The term “science literacy” or “scientific literacy,” which stands for “what the general public ought to know about science” (Durant, 1994), is relevant but not accurate and is usually considered synonymous to the “public understanding of science” (Laugksch, 2000). It is argued that four components of scientific literacy are most likely to help individuals identify misinformation: (1) understanding of scientific practices; (2) identifying and judging appropriate scientific expertise, (3) epistemic knowledge, and (4) dispositions and habits of mind, such as open‐mindedness. However, three of these four components are not commonly used in definitions of scientific literacy (Sharon & Baram-Tsabari, 2020). In other words, science or scientific literacy cannot explicitly capture views of scientific information. Therefore, in terms of individual’s discernibility of science-related misinformation during the COVID-19 pandemic, a comprehensive literacy that integrates the overlapping concept of both scientific literacy and information literacy as a whole is required.

Accordingly, we defined scientific information literacy (SIL) as the ability to practice critical thinking based on scientific evidence, reasonable analysis, as well as the consensus of scientific community, which leads to recognizing science-related misinformation. In particular, during the COVID-19 pandemic, an overarching framework of SIL highlights three fundamental aspects: (1) trust in science. Science plays the unique role in modern society for providing citizens with information that is justified beyond their own observations. That is, nonexpert audiences should routinely defer to scientific judgment and make policy choices that are consistent with evidence-based consensus within the scientific community (Scheufele & Krause, 2019). Thus, identifying misinformation is often a matter of trust. For reliable knowledge about contentious scientific issues during the pandemic, one should turn to science and scientists (Sharon & Baram-Tsabari, 2020). (2) Evaluation of information. It is consistent with the core meaning of information literacy discussed above. (3) Understanding the uncertainty of science. Spreading doubts by referring to the uncertainty of scientific conclusions is a very popular strategy for misinforming the populace (Lewandowsky et al., 2012). Moreover, the risk of scientific uncertainty affecting public trust is magnified for COVID-19, which is defined by uncertainty as a novel coronavirus (Kreps & Kriner, 2020). Therefore, lay audiences should understand that uncertainty is an inherent aspect of science.

Based on this type of literacy, we can measure the extent during the pandemic to which lay audiences identify information that is in line with evidence-based consensus within the scientific community for defeating the attendant “infodemic.”

### PES and SIL

Several studies have revealed that certain types of literacy were associated with public engagement. Some scholars and educators assumed that media literacy could inspire increased engagement and activities in political and civic life for young learners (Ashley et al., 2017; Valenzuela et al., 2019). Valenzuela (2019) demonstrated a paradox that increased political engagement is correlated with increased spread of misinformation. The main reasons are politically engaged users may be more exposed to misinformation, and tend to share misinformation to intentionally deceive others or debunk it. When it comes to PES, it can help not only address controversial scientific issues and construct social consensus but also facilitate science literacy through ISE. For example, PES in ISE allows “scientists and publics to voluntarily participate in lifelong learning in science and to achieve fluency with topics that emerge after they complete formal schooling by exploring ideas, examining current societal issues, challenging the claims of others, and developing their own understanding of science and its relationship with their lives” (McCallie et al., 2009). Though PES in ISE has the potential to foster the type of science literacy, the impact of PES on individual SIL particularly immunity against misinformation remains under-examined. Based on the above discussion, the second hypothesis is proposed as follows:

#### Hypothesis 2 (H2)

PES activities (a) and PES attitudes (b) will positively predict SIL.

**Fig. 1:**
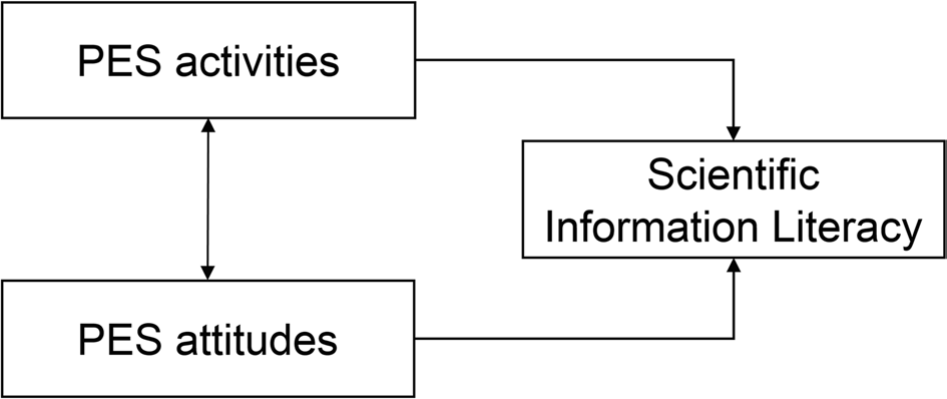
The proposed model of the current research

In brief, by combining the aforementioned hypotheses, Fig. 1 summarizes the framework.

## Method

### Participants and Sampling

This study used a cross-sectional design and was conducted in two Chinese universities, one of which is located in Beijing in North China and the other in Xiamen in South China, from September 24 to November 6, 2020. We employed cluster sampling to construct the study sample. University students studying in the two universities were recruited to complete a questionnaire via an electronic link distributed by each class teacher. To avoid data duplication, each internet protocol address was allowed to open the questionnaire once only. It took approximately 20 to 30 min for participants to complete the questionnaire. All participants were informed of the purpose and procedures of this survey before completing the questionnaire and were informed that they had the right to withdraw from the survey at any time.

A total of 9854 participants participated in the survey. We excluded 1779 samples due to incomplete information or false answers on the lie detection items, with a completion rate of 81.9%, leaving the final sample size in the present study was 8075.

### Measures

#### Sociodemographic Measures

The questionnaire included some self-made items and some items derived from previous studies to measure the interested variables. All original English items were translated into Chinese and back translated by two bilingual researchers.

Sociodemographic measures included a range of sociodemographic information, including gender, age, ethnic group, type of residence, family economic status, perceived social class, and preferred media type. Gender identity was measured by a single question: “Which of the following best describe your gender?” Responses were classified into three categories: male, female, and others. Ethnic group was measured by a single question and responses included two categories: Han ethnic and others. The type of residence was classified into three responses (1 = city; 2 = town; 3 = country). Family economic status was measured by a single question and responses ranged from 1 (very rich) to 5 (very poor). Subjective social class was assessed by an 11-step ladder figure. Participants were asked to choose the step they were currently on. Responses ranged from 0 (the lowest step) to 10 (the highest step). Media preference was measured by a single question: “In terms of information acquisition, do you rely more on traditional media such as TV and newspaper, or new media such as the internet?” Responses were rated on a 5-point scale ranging from 1 (totally rely on traditional media) to 5 (totally rely on new media).

### PES

PES was measured based on previous studies of public participation in genomics research conducted in the Netherlands (Dijkstra et al., 2012) and media use in scientific reports (Jin, 2018). The items were adapted in the present study for specific study purpose. Based on theoretical framework, PES included two main components: PES activities and PES attitudes. The final scale comprised of 13 items on a five-point Likert scale. Respondents were asked to indicate the degree of agreement of below statements from 1 (strongly disagree) to 5 (strongly agree). For instance, “Even as a layperson, I am willing to have dialogs with scientists on scientific issues.” Participants were also asked to indicate the frequency of media use on a five-point scale (1 = not at all; 5 = extremely). For example, “Do you ever read scientific information on TV?” Two corresponding factors (PES activities and PES attitudes) were confirmed by exploratory factor analysis (EFA). The two subscale scores were added separately and a mean score was generated for each subscale.

### SIL

We developed a SIL scale based on previous studies referring to scientific (Miller, 1998; Bybee et al., 2009) and information literacy (Fujii, 2007; Ashley et al., 2013). This scale included five items and participants were asked to respond on a five-point Likert scale (1 = strongly disagree; 5 = strongly agree), assessing the degree of trust in science, evaluation of information, and understanding on the uncertainty of science during the pandemic. Example items includes “To judge whether a claim is scientific information or a rumor, I will collect and analyze more information to draw a conclusion” and “Science is the best way to fight the COVID-19 pandemic.” We examined the construct validity of this scale by exploratory factor analysis (EFA).

### Data Analysis

First, descriptive statistics for sociodemographic variables was calculated. Second, an EFA was performed to explore the construct validity of the PES questionnaire and SIL questionnaire, by using the principal component analysis and direct oblimin rotation. Third, Pearson correlations were conducted to explore the bivariate relationships between measured variables. Fourth, a paired *t* test was performed to examine H1. Finally, a hierarchical linear regression model was constructed to examine H2. We entered variables in separate steps to test the incremental effect size of *R*^*2*^ in each step and used the ordinary least squares (OLS) method. Sociodemographic variables and media usage preference were entered in Step 1, followed by PES activities and PES attitudes in Step 2. All data analyses were performed using SPSS version 23.0, and data visualization using R version 4.0.2. Statistical significance level was set at two-sided 0.05 in the present study.

## Results

### Sociodemographic Statistics

We included the 8075 participants in the final sample. The age range was 17–40 years old (*M* = 20.81, SD = 3.69), and 59.6% of females made up for the sample. The majority of the participants were Han Ethnic (89.1%), urban dwellers (85.1%) and with moderate family economic status (74.9%). Social class was measured with an 11-step ladder from 0 at the lowest and 10 at the highest step. Most participants reported their social class as Step 3 to 6 (78.9%), and preferred to use new media rather than traditional media to access information (79.6%).

### EFA of Selected Items

The total scores of PES (13 items including PES activities and PES attitudes) ranged from 13 to 65, with 0% scored 13 and 0% scored 65 (both less than 15%), which showed that the PES questionnaire did not demonstrate ceiling or floor effects (Lim et al., 2015). Results of Kaiser–Meyer–Olkin (KMO) Measure of Sampling Adequacy test and the Bartlett Test of Sphericity showed that the data was suitable for EFA (KMO = 0.81; *χ*^2^ = 29,069.34, *df* = 78, *p* < 0.001). In order to be consistent with two theoretical components of PES (i.e., PES activities and PES attitudes), a fixed two-factor model was performed. The extracted two factors accounted for 44.42% of the total variance. Table 1 shows the corresponding items and factor loadings for each item. The PES score was calculated by adding up all items (Cronbach’s *α* = 0.81). Furthermore, the composite scores for PES activities (Cronbach’s *α* = 0.82) and PES attitude (Cronbach’s *α* = 0.67) were generated, with higher scores indicating higher level of PES activities or more positive attitudes toward PES.

**Table 1:** Factor loadings of the PES questionnaire (*N* = 8075)

	Items	Factor 1	Factor 2
1	Do you ever read scientific information on books and publications?	0.76	
2	Do you ever read scientific information on TV?	0.75	
3	Do you ever read scientific information on the internet?	0.71	
4	Do you ever read scientific information on WeChat?	0.69	
5	Do you ever hear scientific information on radio?	0.66	
6	How interested are you in science and technology reports or topics?	0.65	
7	Do you ever read scientific information on blog or Weibo (Chinese Twitter)?	0.60	
8	Do you ever attend a science-related conference or participate in academic activities?	0.48	
9	Do you ever participate in any science-related social activities, such as expressing your opinion for or against genetically modified food?	0.32	
10	My participation in science communication has positive effects on scientific research		0.80
11	My participation in activities that disseminate scientific knowledge, methods, processes, or practices can influence governmental decisions concerning the scientific issue		0.74
12	Even as a layperson, I am willing to have dialogs with scientists on scientific issues		0.68
13	Many socio-scientific issues are public issues that require public participation in decision-making		0.55

*PES* public engagement with science. Factor 1: PES activities; Factor 2: PES attitudes. Factor loadings less than 0.3 were omitted

Similar to the PES questionnaire, the construct validity of SIL questionnaire was confirmed by EFA. No ceiling or floor effects were found (0% scored 5 and 4.2% scored 25, both less than 15%), indicating appropriate item difficulty of this questionnaire. KMO and Bartlett Test of Sphericity also showed that the data was suitable for EFA (KMO = 0.74; *χ*^2^ = 4956.82, *df* = 10, *p* < 0.001). Based on the criterion of Eigenvalues greater than 1, one factor was extracted, accounting for 42.44% of the total variance. Table 2 shows the results of EFA and corresponding items. All item scores were added to generate a composite score for each participant (Cronbach’s *α* = 0.65), with higher scores indicating higher levels of SIL.

**Table 2:** Factor loadings of the SIL questionnaire (*N* = 8075)

	Items	Mean ± SD	Loading coefficient
1	Science is the best way to fight the COVID-19 pandemic	4.09 ± 0.74	0.71
2	I trust scientists more than other social groups during the COVID-19 pandemic	3.96 ± 0.66	0.71
3	Regarding the information about the COVID-19 pandemic, I hope to see the interpretation of authoritative scientists	4.43 ± 0.57	0.67
4	To judge whether a claim is scientific information or a rumor, I will collect and analyze more information to draw a conclusion	4.09 ± 0.66	0.60
5	Many controversial issues related to science during the COVID-19 pandemic are due to the uncertainty of science itself, for example, scientists’ limited knowledge of the virus	3.85 ± 0.76	0.55

*SIL* scientific information literacy

### Correlations Between Interested Variables

**Fig. 2:**
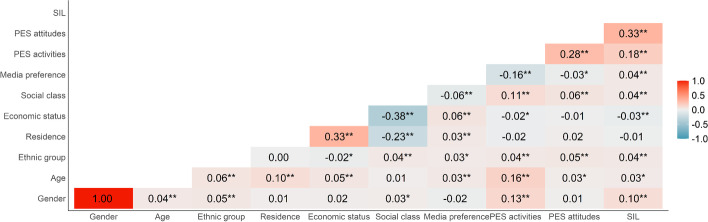
Pearson correlations between variables. Gender and ethnic group are dummy variables. Male = 1, others = 0; Han = 1, others = 0. *p < 0.05, **p < 0.01

The results of Pearson correlations between sociodemographic and variables of interest showed that significant correlations were found between sociodemographic variables, independent variables and SIL (see Fig. 2). The results showed that correlations between sociodemographic characteristics and SIL were small (*r* < 0.30). Moreover, SIL was positively correlated with both PES activities (*r* = 0.18, *p* < 0.001) and PES attitudes (*r* = 0.33, *p* < 0.001).

### Differences Between PES Activities and PES Attitudes

**Fig. 3:**
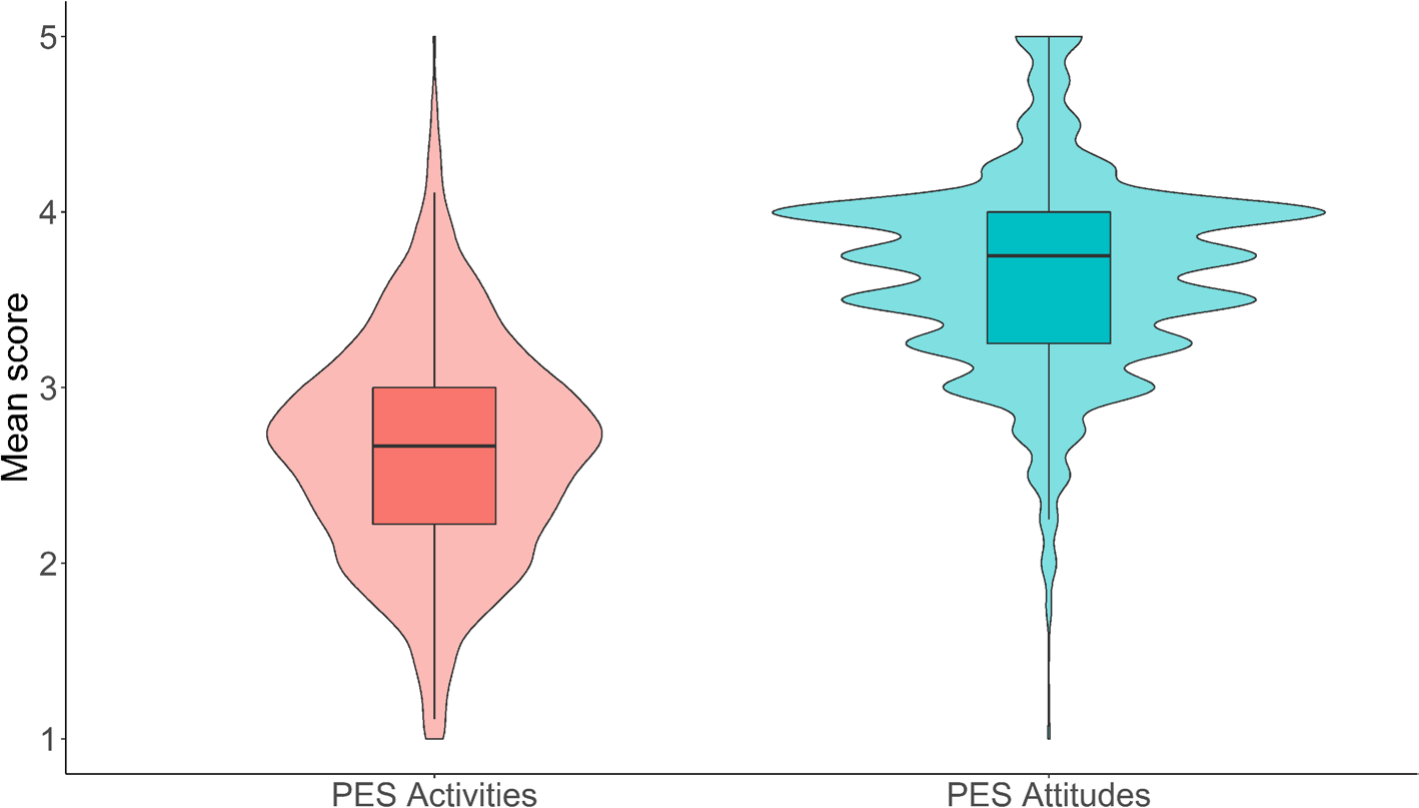
Mean scores for PES activities and PES attitudes in China

We hypothesized that Chinese public showed significantly lower levels of PES activities than their attitudes toward PES (H1). The paired *t* test results supported H1 (see Fig. 3). Individual attitude toward PES (*M* = 3.65, SD = 0.55) was significantly higher than their actual activities of that (*M* = 2.64, SD = 0.62; *t* (8074) = 128.43, *p* < 0.001, *d* = 1.36, 95% CI [1.323, 1.392]), indicating the effect size of the difference is acceptable.

### Effects of PES Activities and PES Attitudes on SIL

We assumed that both PES and attitude toward PES would positively predict SIL. We constructed a hierarchical linear regression model by two steps (see Table 3). The multicollinearity test showed that tolerance values were higher than 0.79 and variance inflation factor values were less than 1.26 for all variables entered in the models (Cohen et al., 2013). In Model 1, sociodemographic characteristics accounted for only 1.4% of the total score of SIL. Males (*β* = 0.10,* t* = 8.77, *p* < 0.001) and those who preferred using new media (*β* = 0.04,* t* = 3.56, *p* < 0.001) were found to be significantly associated with higher levels of SIL. In Model 2, controlling for sociodemographic variables, the increment of effect size was significant (*F* (9, 8065) = 131.97, *p* < 0.001, *△R*^*2*^ = 0.11). Both PES activities and PES attitudes were found positively associated with SIL, especially the PES attitudes. The more active PES attitudes (*β* = 0.30,* t* = 27.85, *p* < 0.001) and the more PES activities are (*β* = 0.09,* t* = 8.27, *p* < 0.001), the higher level of SIL is. The results of Model 1 and 2 supported H2 (a) and H2 (b).

**Table 3:** Hierarchical linear regression coefficients for scientific information literacy (*N* = 8075)

Variables	Model 1	Model 2
	*β* (SE)	*β* (SE)
Gender (male)	0.10*** (0.05)	0.09*** (0.05)
Age	0.02* (0.01)	0.00 (0.01)
Ethnic group (Han)	0.03* (0.08)	0.01 (0.07)
Residence	− 0.00 (0.04)	− 0.01 (0.04)
Economic status	− 0.02 (0.05)	− 0.03* (0.05)
Social class	0.03*(0.02)	− 0.01 (0.02)
Media preference	0.04***(0.03)	0.06*** (0.03)
PES activities		0.09*** (0.00)
PES attitudes		0.30*** (0.01)
*R*^*2*^	0.014	0.128
*△R*^*2*^	0.014***	0.114***

Gender and ethnic group are dummy variables. Male = 1, others = 0; Han = 1, others = 0. All *β* are standardized regression coefficients

*p < 0.05

**p < 0.01

***p < 0.001

## Discussion

The COVID-19 outbreak brought out the “infodemic,” which has been a huge challenge to human beings globally in terms of information judgment and discernment, especially for science-related controversies. Based on a cross-sectional study conducted in China during the COVID-19 pandemic, this study investigated the effects of PES on SIL.

First, this study found that levels of PES attitudes are significantly higher than PES activities. That is, people have high expectations and strong intention of PES; however, in China, the actual participation in science is not as positive as their attitude. This discovery can help us better understand the specific context of PES in China especially in respect of the COVID-19 pandemic. It indicated that although the PES model is not perfect, the idea of public participation has been rooted in China. The deficit model should be replaced by the PES, which emphasizes public participation and encourages dialog between the lay public and scientific community. Moreover, the lower level of PES activities shows that some structural factors are hindering the construction of a scientific public sphere in China. Thus this transformation seems to be urgent in China.

Second, this study revealed that both PES attitudes and PES activities positively predicted SIL. That is, boosting public enthusiasm and expectations for engagement with science or providing more opportunities to participate in science-related activities would promote individual SIL. As previous studies (Jones-Jang et al., 2019; Guess et al., 2020) have confirmed the effect of media literacy intervention on misinformation discernment, our result is of great significance for combating the “infodemic” caused by the COVID-19 pandemic. Considering the lower levels of PES activities in China discussed above, we should take action immediately. For example, as the most important official institution of science communication and ISE in China, the China Association for Science and Technology (CAST), should understand the necessity of PES and regard it as a strategy for improving individual SIL. Accordingly, in major public issues related to science such as the COVID-19 vaccination, CAST must attempt to hold scientific consensus conferences, science communication forums, and other better-informed and more balanced public debates to promote PES. Moreover, a diverse set of actors including scientists, policy makers, citizens, and interest organizations should be involved in PES, providing more plural forms of public knowledge (Van Est, 2011).

Third, as the first rigorous empirical research demonstrating that PES has a positive effect on individual SIL, this finding has contributed to deepening the understanding of PES in the context of ISE. On the one hand, PES can be understood as a learning process focused on growth in mutual understanding, awareness, and knowledge of competing perspectives on socio-scientific issues as well as “facts.” PES thus is positioned as providing opportunities for empowering individuals for further involvement in decision making, policy deliberation as well as learning (Lehr et al., 2007). On the other hand, “thinking like a scientist” is better than just “knowing lots of science” for the lay public, especially in face of the “infodemic.” As our SIL scale consists of measures on the degree of trust in science, evaluation of information, and understanding on the uncertainty of science, PES can be used not only to rebuild public trust in science as a science communication mode but also to promote individual information discernibility and foster scientific way of thinking. The paradigm shift from deficit model to PES has proved that PES has been the new “royal road” to rebuild public trust (Bauer et al., 2007). Moreover, PES may work as a message interpretation or education process which mediates the relationship between exposure to misinformation and subsequent decision making (Jones-Jang et al., 2019). Furthermore, PES can develop scientific literacy as continuing ISE at all stages of the life cycle (Bauer et al., 2007).

Fourth, the results showed that sociodemographic characteristics were predictive on individual SIL. For example, males, those preferred using new media, were associated with higher levels of SIL. Moreover, the preference type of media use was found to predict SIL significantly during the pandemic. The new media preference is associated with higher levels of SIL. Previous studies have explored digital media literacy as a core competency for engaged citizenship in a participatory democracy (Mihailidis & Thevenin, 2013). In an age of increased reliance on digital and social media, citizens who prefer new media tend to have better access to democratic participation that coincides with the discussion above. It is notable that the effect size of these sociodemographic variables was much smaller than PES variables.

## Limitations and Implications

Although this study is one of the first investigations of PES in China during the pandemic, it has some limitations. First, the participants were primarily university students, who are not representatives of the entire population of Chinese citizens. Future studies could employ participants of all ages to testify and generalize the conclusion. Second, we assumed that education level might be an important variable in promoting SIL. It is a pity that the education level did not vary enough due to the all-student sample in this study. Future research could involve the education level as a SIL-related variable. Third, the reliability of SIL is not very high. We assume that cultural difference may induce the invariance in the reliability. We believe that SIL may cover more contents than we measured, and a more comprehensive and sensitive scale should be developed to measure SIL in the future. Fourth, we used a cross-sectional design, which was limited in causal inference; thus, laboratory behavioral experiments and longitudinal studies are necessary to explore causal explanations in future studies. Finally, the total effect size of PES was not high, indicating that there are many other factors influencing SIL. Future studies should include more possible factors relating to SIL in the theoretical framework.

Regardless of these limitations, the present study provided critical theoretical and practical implications. In theory, previous studies have focused on beneficial effects of the public understanding of science on individual scientific literacy and PES on individual attitudes toward science (Stilgoe et al., 2014). Therefore, the relationship between PES and literacy has always been neglected. The present study has highlighted the potential advantages of PES because it is conducive to not only scientific decision-making and the resolution of science-related disputes but also enhancing individual immunity to misinformation.

In practice, apart from regulating producers and distributing platforms, such as social media sites, SIL education is considerably crucial in helping audiences to develop the ability to better manage and understand scientific information (Jones-Jang et al., 2019). Although media literacy initiatives are typically expensive to develop, slow to roll out, and reactive rather than proactive (Linden et al., 2020), the present study provided an effective audience-centered approach to develop SIL. Because the creation and diffusion of misinformation are easier than ever during the COVID-19 pandemic, we must promote PES as a novel and thorough type of informal education on SIL.

## Data Availability

The data of this study are available from the corresponding author on reasonable request. The data are not publicly available due to privacy and ethical restrictions.
